# Tuberculous Disease of the Knee Joint—II

**Published:** 1908-10-10

**Authors:** 


### TUBERCULOUS DISEASE OF THE KNEE JOINT—II.

In a previous article the early signs and symptoms of this condition were described. In a case which is not treated at all, or is improperly treated, the condition goes from bad to worse, the patient being finally reduced to a state in which he becomes completely bedridden because progression is impossible. It is said that this happens sooner in cases in which the bone is primarily attacked; but wherever the disease starts, caries of the articular end of the bone occurs sooner or later, the whole synovial cavity becomes converted into a bag of pus; and periarticular abscesses form which come to the surface, discharge their contents, and leave peculiarly intractable sinuses, especially if, as often happens, they become infected secondarily with ordinary pyogenic micro-organisms.

In the late stages the leg becomes deformed in a typical manner. It is flexed at the knee, the whole tibia is displaced backwards on the femur, and is also externally rotated. This is known as "the triple displacement." It is due to over-action of the biceps femoris, which the weakened ligaments of the joint cannot resist. It might be thought at first sight that the quadriceps extensor would be a more powerful muscle than the biceps, but this is not actually the case: it will be found as a general rule that, when a limb is diseased, the flexor groups of muscles tend to overcome the extensors.

The operative treatment of a tuberculous joint is wholly unsatisfactory; even if the operation itself is a success and the objects which the surgeon has in performing it are attained, the result is not one of which we can justly be proud. At the best the patient is left with an ankylosed leg some inches shorter than the sound one; a leg which is an imperfect means of progression and an inadequate column of support. Of course cases are only too frequently seen which have been allowed to go so far that the condition depicted above has made its appearance, and in these surgical interference is absolutely necessary to save the limb.

But unless this is the case, every attempt should be made to arrest the disease by palliative measures, and to preserve a useful limb for the patient. The moment tuberculous disease of the knee is suspected, absolute rest must be enjoined. Those who have read Hilton's classical "Lectures on Rest and Pain" will appreciate the enormous benefit which rest, and rest alone, can produce in surgical tuberculosis. It is insufficient merely to put the patient to bed; the limb must also be put in some form of retentive apparatus. In the case of a tuberculous knee, in which the joint is flexed, it is well to keep the leg on a McIntyre splint which can be adjusted to a suitable angle. The flexion in the early stages is not due to the same cause as the triple displacement which appears later. The knee is slightly bent, because this is the position of greatest comfort if the synovial membrane contains fluid or is diseased. This is the case in almost all diseases of the knee-joint. If a patient comes up complaining of pain in the knee-joint, which is held completely extended, the chances are that he has nothing serious the matter with it. The flexion is best overcome by the application of an extension to the leg, which is applied with strapping in the ordinary way This serves two purposes. It not only overcomes the contraction of the periarticular muscles and tendons, and so gradually gets rid of the deformity, but it also diminishes the starting pains at night, which are a source of great trouble because they prevent the patient getting any proper sleep. It has already been explained that these pains are due to the moving of the infected articular surfaces, one upon the other, when the muscles become relaxed during sleep, and to the subsequent sudden contraction of the muscles in an attempt to keep the surfaces at rest. The extension will effectually prevent this from happening, because in the first place the surfaces are unable to move, and in the second the muscles cannot contract. In an ordinary case it is only a question of time for the extension to overcome the tonic contraction of the periarticular muscles, and the limb will soon be completely extended if the angle of the splint be altered daily, or every other day. For this reason it will be found that a McIntyre's splint will most suitably meet the needs of the case. When the limb is straight, the patient must still be kept prone and the knee absolutely at rest, until all signs of active disease have disappeared. This treatment, combined with a generous diet of easily digested food and, as far as it can be managed, a fresh-air existence, will eventually effect a cure, as far as tuberculosis can ever be called cured. At the best it means that it has been induced to become quiescent. When this stage arrives, the patient can get about with a Thomas's knee-splint for a period which will necessarily vary in each individual case; but as a general rule it should be worn for at least a year, and the longer it can be tolerated the better.

This line of treatment, though attended with success in the long run, is of course tedious, and several methods have been recently suggested with a view to hastening the cure. Von Bier, starting from the observed fact that pulmonary phthisis is rare in mitral stenosis, because, supposedly, the lung is in a state of chronic venous congestion, has proposed to produce the same condition artificially by constricting the venous circulation in the affected limb. A piece of lint is applied round the limb above the joint, and on top of this an elastic bandage is applied with just sufficient pressure to compress the veins. The method is not without pain, and at the beginning the patient can only tolerate its application for a short time. When, however he gets more accustomed to it, it can be left in position for as long as eighteen hours out of the twenty-four. The method has been tried on a number of cases at most of the London hospitals, but the general consensus of opinion does not seem to be in favour of it. It is distinctly painful, without producing any proportionate benefit; and, further, the cases which apparently improved under it, nearly all relapsed later.

*(To be continued.)*

